# Differentiations of Chitin Content and Surface Morphologies of Chitins Extracted from Male and Female Grasshopper Species

**DOI:** 10.1371/journal.pone.0115531

**Published:** 2015-01-30

**Authors:** Murat Kaya, Evaldas Lelešius, Radvilė Nagrockaitė, Idris Sargin, Gulsin Arslan, Abbas Mol, Talat Baran, Esra Can, Betul Bitim

**Affiliations:** 1 Department of Biotechnology and Molecular Biology, Faculty of Science and Letters, Aksaray University, 68100, Aksaray, Turkey; 2 Aksaray University, Science and Technology Application and Research Center, 68100, Aksaray, Turkey; 3 Department of Biology, Vytautas Magnus University, 44404 Kaunas, Lithuania; 4 Selcuk University, Faculty of Science, Department of Chemistry, 42075, Konya, Turkey; 5 Selcuk University, Faculty of Science, Department of Biochemistry, 42075, Konya, Turkey; 6 Guzelyurt Vocational School, Aksaray University, Guzelyurt, Aksaray, Turkey; 7 Department of Chemistry, Faculty of Science and Letters, Aksaray University, Aksaray, Turkey; Institute of Plant Physiology and Ecology, CHINA

## Abstract

In this study, we used Fourier transform infrared spectroscopy (FT-IR), elemental analysis (EA), thermogravimetric analysis (TGA), X-ray diffractometry (XRD), and scanning electron microscopy (SEM) to investigate chitin structure isolated from both sexes of four grasshopper species. FT-IR, EA, XRD, and TGA showed that the chitin was in the alpha form. With respect to gender, two main differences were observed. First, we observed that the quantity of chitin was greater in males than in females and the dry weight of chitin between species ranged from 4.71% to 11.84%. Second, using SEM, we observed that the male chitin surface structure contained 25 – 90nm wide nanofibers and 90 – 250 nm nanopores, while no pores or nanofibers were observed in the chitin surface structure of the majority of females (nanofibers were observed only in *M. desertus* females). In contrast, the elemental analysis, thermal properties, and crystalline index values for chitin were similar in males and females. Also, we carried out enzymatic digestion of the isolated chitins using commercial chitinase from *Streptomyces griseus*. We observed that there were no big differences in digestion rate of the chitins from both sexes and commercial chitin. The digestion rates were for grasshoppers’ chitins; 88.45–95.48% and for commercial chitin; 94.95%.

## Introduction

Scientists have focused on chitin isolation and characterization from crabs, shrimps, crayfish and mushrooms [1–4]. In recent years, researchers have studied chitin extracted from insects, anthozoans, sponges and small crustaceans (*Oniscus asellus, Asellus aquaticus* and *Gammarus argaeus*) [5–10].These studies mentioned works which were focused on chitin characterization, content evaluation, physicochemical, and the investigation of functional properties. However, some studies were conducted to compare the chitin contents of two components (stipe and pileus) of mushrooms and to compare the chitosan physicochemical and functional properties isolated from crab shells harvested in three different years [11, 12]. Until now, no studies have been conducted on chitin isolation and the characterization in terms of gender (female and male).

Chitin (C_8_H_13_O_5_N)n is a natural polysaccharide and is the second most abundant biopolymer after cellulose. Chitin is a long-chain polymer composed of (1–4)-linked 2-acetamido-2-deoxy-b-D-glucose and was isolated from the cell walls of mushrooms for the first time in 1811 by Henri Braconnot [13, 14]. Generally, chitin is found in the exoskeletons of arthropods (crustaceans, insects, myriapods and arachnids), also in the cell structure of algae and yeast, and in the cell walls of fungi [7, 15–17]. Naturally, chitin is found in three crystalline polymorphic forms. Within each form there are different orientations of the microfibrils: α-chitin has antiparallel chains, β-chitin has parallel chains, and γ-chitin has the mixture of parallel and anti-parallel chains [15, 18].

Chitin and chitin-derived products are attracting great interest because of their wide range of potential applications within biotechnology, medicine and pharmacology, agriculture, cosmetics, and wastewater treatment [14, 16, 19, 20]. Chitin and its derivatives have a wide range of useful biological properties such as non-antigenicity, biocompatibility, biodegradability and non-toxicity [21, 22]. Chitin and its products, mostly chitosan, are functional polysaccharides and their potential applications within various fields are being actively investigated [7].

Recently chitin and its derivatives have found wider applications (i.e., polyelectrolyte properties, gel-forming ability, high adsorption capacity, healthy weight loss pills, and wound healing applications, matrix for immobilization of biomolecules, support for biosensors, heavy metal removal, and removal of radioactive waste) [14, 16, 19, 22]. This has gained attention of many researchers and made them search for new chitin sources. Crab, shrimp and crayfish have been preferred for commercial production of chitin but new chitin sources like insects could be exploited.

Orthoptera is the order of insects that includes grasshoppers, crickets, locusts, katydids and their related species. Grasshoppers are widespread throughout the world [23]. Approximately 20 000 described orthopteran species have been acknowledged worldwide up to now [24], but these organisms have been highly ignored with regards to chitin structure.

The aim of this study is to reveal the characterization differences in chitin structures isolated from the female and male of the four orthopteran species. The percentage chitin content of grasshoppers was recorded and the physicochemical properties of chitins were determined by FT-IR, elemental analysis, TGA, XRD and SEM. Also, we studied enzymatic (chitinolytic) digestion of the chitins to reinforce the analyses by the aforementioned characterisation tools.

## Material and Methods

### Material

Localities of the species are shown in Table 1. No specific permissions were required for these locations. The field studies did not involve endangered or protected species and provide the specific location of your study. The GPS coordinates:

**Table 1:** Localities of the grasshopper species collected from Turkey.

*Celes variabilis*	Çorum Beydili-Çatak Village 2005 (Coordinates: 40°41' N and 34°50'E)
*Decticus verrucivorus*	Artvin Şavşat 2006 (Coordinates: 41°15' N and 42°21' E)
*Melanogryllus desertus*	Samsun 19 Mayıs University Campus 2003 (Coordinates: 41°22' N and 36°12' E)
*Paracyptera labiata*	Kastamonu, Tosya 2005 (Coordinates: 41° 1' N and 34° 2' E)

1- Çorum Beydili-Çatak Village 2005 (Coordinates: 40°41' N and 34°50'E).

2- Artvin Şavşat 2006 (Coordinates: 41°15' N and 42°21' E).

3- Samsun 19 Mayıs University Campus 2003 (Coordinates: 41°22' N and 36°12' E).

4- Kastamonu, Tosya 2005 (Coordinates: 41° 1' N and 34° 2' E).

Female and male of the species were collected in the same location. Laboratory samples collected in 2003, 2005 and 2006 were used for chitin extraction in this study.

### Chitin Extraction

The grasshoppers used for this experiment firstly were dried in an oven at a temperature of 50 °C for 4 days. Later, dried grasshoppers were milled to a powder and then weighed.

The chitin extraction from grasshoppers was followed as described by Kaya et. al. [9] with small changes. Briefly, the powder was treated with 4 M HCl solution (50 mL) at 75 °C for 2 hours to remove minerals and catechols. Later on, the sample was filtered off and the residue was rinsed thoroughly with distillate water. To achieve deproteinization step, the filtrate was treated with 50 mL of 4 M NaOH solution for 20 h at 150 ºC. The mixture was filtered off and washed repeatedly with distillate water. Finally, the samples were placed in an oven at 60 °C for 24 hours to be dried. After all, dry weight of chitin contents was calculated.

Additionally isolation chitin samples were led to air-dry in shade at room temperature for 10 days. Then dried samples were weighted and chitin content of each male and female grasshopper species were determined.

### Fourier Transform Infrared Spectroscopy (FT-IR)

Extracted chitins from female and male grasshoppers were analysed a Perkin Elmer mark FT-IR Spectrometer at 4,000–625 cm^-1^.

### Elemental Analysis (EA)

Elemental analysis was conducted by Elemental analyzer Flash 2000, to ascertain the % C, N and H contents of the chitins. The degree of acetylation (DA) was calculated using the formula 1 [25]:

DA=[(C/N–5.14)/1.72]x100 (1)

### Thermogravimetric Analysis (TGA)

To obtain TG and DTG curves, chitins isolated from grasshoppers were analysed with EXSTAR S11 7300 at a heating rate of 10 °C per min^-1^.

### X-ray Diffraction (XRD)

A RigakuDmax 2000 was used to obtain X-ray diffraction peaks of chitins isolated from grasshoppers female and male. The data of analyse was obtained at 40 kV, 30 mA and 2θ with the scanning angle from 5 to 45°. Crystalline index values (CrI) of chitins were calculated according to Formula 2 (below) [26]:

$${\text{CrI}}_{110}=\;\left[\left(I_{110}–I_{\text{am}}\right)\;/I_{110}\right]\;\text{x}\;100$$ (2)

I_110_ is the maximum intensity at 20 ≈ 19°. I_am_ is the intensity of amorphous diffraction at 20 ≈ 13°.

### Scanning Electron Microscopy (SEM)

Chitin samples extracted from female and male were coated with gold by using “Sputter Coater (Cressingto Auto 108). After gold covering, chitin samples were analysed via QUANTA- FEG 250.

### Chitinase Hydrolytic Activity on the Isolated Chitins from Both Sexes and Commercial Chitin

Commercial chitin (shrimp shells, pcode: 1001416772), chitinase (chitinase from *Streptomyces griseus*, EC. 3.2.1. 14), Na_2_CO_3_, NaH_2_PO_4_ and Na_2_HPO_4_, HCl and ethanol was purchased from Sigma-Aldrich. Potassium hexacyanoferrate(III) was obtained from Merck.

Colloidal chitin (1% w/v) as a substrate for enzymatic hydrolysis of chitin was prepared as described elsewhere [27]. Briefly, 100 mg of chitin (commercial chitin and grasshoppers’ chitins) was mixed with 2 mL of cold HCl acid solution (37%). The mixture was placed at 4 °C for 24 h to arrest hydrolysis of chitin. Then, 5 mL of water-ethanol mixture (50:50 v/v) was poured with rapid stirring. The mixture was dialysed against first water then sodium phosphate buffer (100 mM, pH: 7.0) for 2 h. The enzyme solution was prepared by dissolving 5 mg of chitinase in 10 mL of phosphate buffer (100 mM, pH: 7.0). The reagent for spectrophotometric determination of the reducing end groups were prepared by dissolving 0.025 g of potassium hexacyanoferrate(III) in 0.5 L of 0.5 M Na_2_CO_3_ solution [28, 29]. The amount of the reducing sugar groups following the enzymatic digestion was determined using potassium ferricyanide assay. Briefly, enzyme solution (1 mL) was added into colloidal chitin (2 mL) solution and then incubated at room temperature for 72 h. The reaction was terminated by boiling for 5 min. After boiling, the mixture was centrifuged at 8000 g for 15 min. The reagent (2 mL) was mixed with the supernatant (2 mL) and placed in boiling water for 20 min. Following the centrifugation (at 8000 g, 15 min), absorbance measurement was performed at 420 nm with a UV-vis spectrophotometer (Shimadzu, Tokyo, Japan, Model 1601) [28]. The amount of the reducing end groups from hydrolysis of chitin was determined colourimetrically from the decrease in the absorbance of ferricyanide.

## Results and Discussion

### Chitin Contents of Female and Male Grasshopper Species

In this study chitin was isolated from female and male of four grasshopper species (*Celes variabilis, Decticus verrucivorus, Melanogryllus desertus, Paracyptera labiata*). Chitin content of female and male are showed in Table 2. Previous studies showed that chitin content in insects is 6–36%, depending on various species [10]. Current study results showed that dry weight of chitin isolated from grasshoppers female and male was 4.71–11.84%. In all species chitin content was higher in male than in female. In *D. verrucivorus* and *P. labiata* chitin content difference was less than 1%, in *C. variabilis* and *M. desertus* difference was around 3%. The highest dry weight of chitin was observed in *D. verrucivorus* male (11.84%). The lowest dry weight of chitin was observed in *M. desertus* female (4.71%).

**Table 2:** Chitin content of female and male grasshoppers.

**Species name**	**Gender**	**%Chitin content (dried at 60 °C)**	**%Chitin content (at shade dried)**
*Celes variabilis*	female	6.65	6.97
	male	9.93	10.21
*Decticus verrucivorus*	female	10.03	10.43
	male	11.84	12.07
*Melanogryllus desertus*	female	4.71	4.96
	male	7.35	7.84
*Paracyptera labiata*	female	6.80	7.23
	male	7.60	8.11

Masses of chitin samples dried at room temperature in shade were relatively higher than the ones that were dried at 60 °C for 24h (4.96–12.04%.). This can be explained by the mass of water molecules absorbed by the samples. Table 2 presents the chitin content obtained two different drying conditions.

### Chitinase Digestive Activity

Chitinase is an endochitinase which cleaves the polymer randomly by hydrolysis of β-1, 4 glycosidic bonds, releasing monomers as N-acetyl glucoseamines or longer segments as chitiobioses, chitotriose and soluble oligosaccharides [30, 31]. Chitio-oligosaccharides intermediates released during hydrolytic enzymatic degradation of colloidal chitins were assayed (Table 3). The table lists the quantification of hydrolytic products from the enzymatic digestion of colloidal chitins in three days incubation. Similar response to chitinase activity was observed for all colloidal chitins. However, chitinase exhibited the highest chitinolytic activity on chitin from *D. verrucivorus* (female) and the lowest from *P. labiata* (female). Chitin digestion rates varied in the range of 88.45–95.48% for grasshoppers’ chitins. The rate for the commercial chitin was 94.95%. We did not observe any correlation between the hydrolysis rates of the chitin substrates from both sexes. Chitinase digestive rates of the grasshoppers’ chitins were found to be close to that of commercial chitin. This indicates that the purity of the grasshoppers’ chitins were nearly identical to the commercial one. The chitin isolation method we employed seems to be appropriate for producing chitin from grasshoppers at commercial standard.

**Table 3:** The reducing sugar assay of chitins from both sexes of grasshopper species: the concentration of reducing intermediates released in enzymatic hydrolysis of colloidal chitins in 72 h incubation, at room temperature.

**Chitin source**	**Reducing ends concentration (µM)**	**Decrease in absorbance (%)**
***Celes variabilis* (female)**	141,56	93,23
***Celes variabilis* (male)**	143,17	94,29
***Decticus verrucivorus* (female)**	144,99	95,48
***Decticus verrucivorus* (male)**	138,53	91,24
***Melanogryllus desertus* (female)**	140,34	92,43
***Melanogryllus desertus* (male)**	135,09	88,98
***Paracyptera labiata* (female)**	134,28	88,45
***Paracyptera labiata* (male)**	141,15	92,96
**Commercial chitin**	144,18	94,95

### FT-IR

In nature, chitin can be found in three different polymorphic forms: alpha, beta and gamma [32]. Most abundant form of chitin in nature is α-chitin, which is found in various arthropod species. Three peaks around 1650, 1620 and 1550 cm^-1^ observed in FT-IR spectrum indicates that exanimated chitin is in alpha form [33–35].

**Figure 1:**
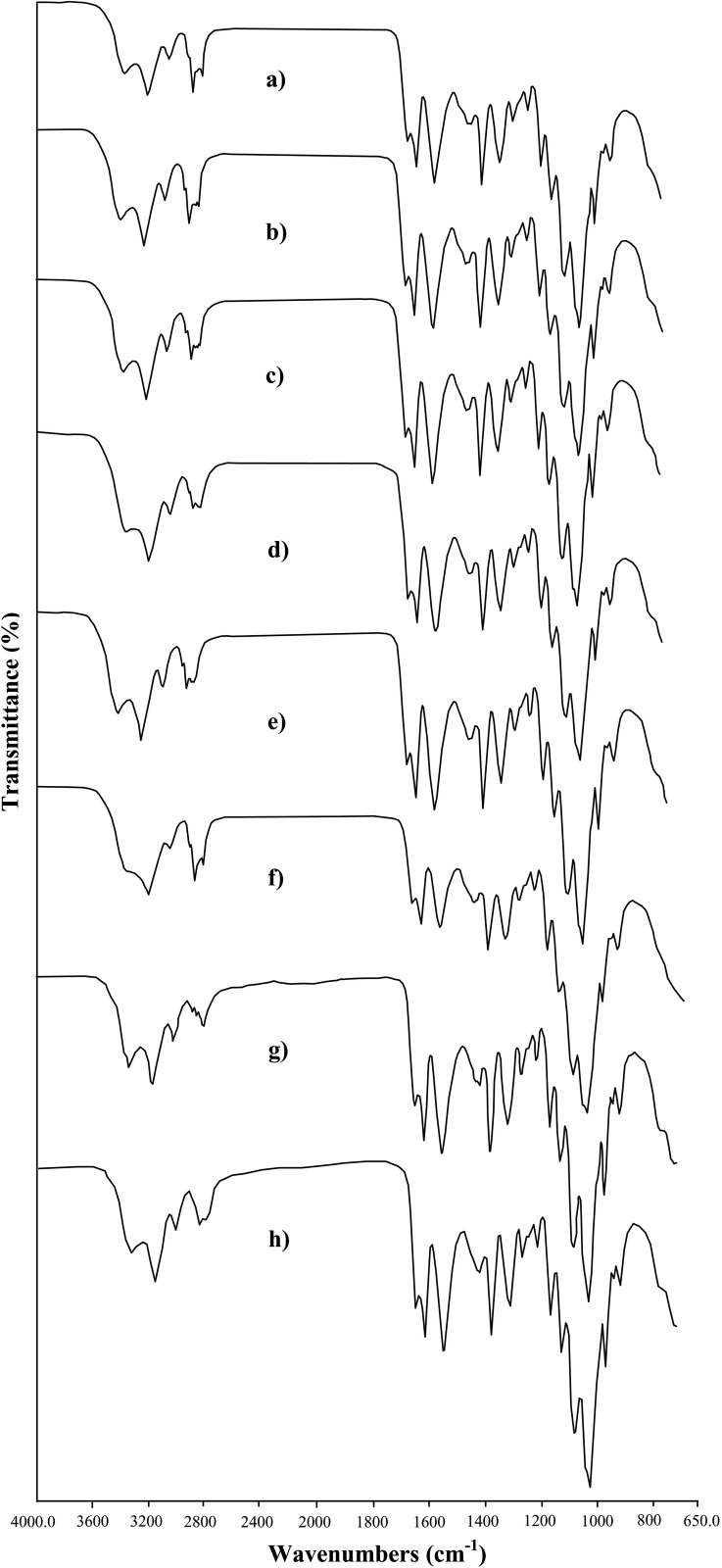
FT-IR spectra of the α-chitin isolated from female and male of five grasshopper species. a) *Celes varabilis* female, b) *C. varabilis* male, c*) Dectius verrucivorus* female, d) *D. verrucivorus* male, e) *Melanogryllus desertus* female, f) *M. desertus* male, g) *Paracyptera labiata* female, h) *P. labiata* male.

FT-IR spectrum of female and male of same species were practically the same and no significant differences were observed. Moreover, no significant differences in chitin structure were perceived after FT-IR analyse between different studied species. In the current study, all exanimated chitin was found to be in α-chitin form. As seen in Fig. 1, we observed three peaks around 1650, 1620 and 1550 cm^-1^, which shows us that isolated chitin is in alpha form. These three peaks were also observed by Paulino et al. [5, 7, 36], and other authors. The absorption peaks at 1540 cm^−1^ were absent. According to Majtan et al. [37] this shows that chitin is protein free.

### EA

Chitin was isolated from four grasshopper species, both female and male, and subjected to elemental analysis. Carbon (C), nitrogen (N) and hydrogen (H) percentages, C/N ratios, and degree of acetylation (DA) are given in Table 4. In this study, the N content of extracted chitin samples was found to be less than 6.89%, which is the theoretical N content for fully acetylated chitin [7]. According to Majtan et al. [37], a lower N content indicates the presence of some residual proteins in chitin. The highest chitin percentage was observed in female *M. desertus* (6.62%), and the lowest in female *C. variabilis* (5.68%).

**Table 4:** Results of elemental analyses (EA) and degree of acetylation (DA).

**Species name**	**N%**	**C%**	**H%**	**C/N**	**DA(%)**
*Celes variabilis* (female)	5.68	46.85	6.62	8.24	180.71
*Celes variabilis* (male)	6.23	45.44	6.31	7.29	125.21
*Decticus verrucivorus* (female)	6.34	45.13	6.37	7.12	115.0
*Decticus verrucivorus* (male)	6.43	45.05	6.56	7.01	108.5
*Melanogryllus desertus* (female)	6.62	48.99	6.86	7.40	131.41
*Melanogryllus desertus* (male)	6.08	48.90	6.88	8.04	168.76
*Paracyptera labiata* (female)	6.17	49.0	6.92	7.94	162.8
*Paracyptera labiata* (male)	6.25	46.10	6.41	7.38	130.0

To determine the degree of purification, DA was calculated from the results of the elemental analysis. Although all chitin isolations were performed using the same method, the DA values varied from 108.5% (male *D. verrucivorus*) to 180.7% (female *C. variabilis*). Sajomsang and Gonil [2] suggested that some inorganic materials may remain in samples with DA values higher than 100%. In our study, in both females and males from all species, the DA values were above 100% (Table 4). These results indicate that some inorganic materials are still present in the chitin structure. However, the purity of chitin in this study is comparable to that observed in other studies. For example, the DA values of chitin isolated from bumblebees, *Oniscus asellus* and shrimps ranged from 132.5% to 237.2% [10, 37].

The results of this study also show that the purification level of isolated chitin is higher in females than in males. Three out of four DA values in females were higher than in males, and only in *M. desertus* were male DA values higher than female.

As seen in Fig. 1, FT-IR spectrum show that there were no significant differences in chitin structure of male and female samples.

### TGA

**Figure 2:**
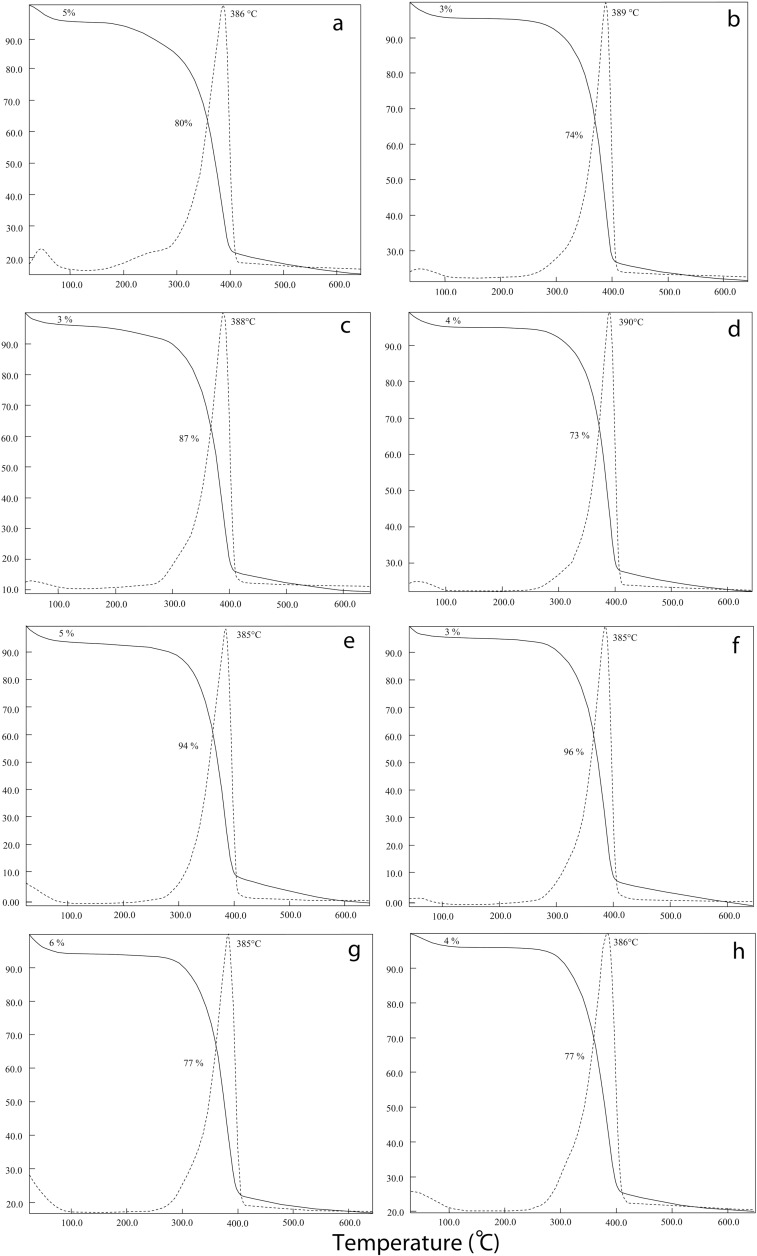
TGA curves of *α*-chitin extracted from female and male grasshoppers. a) *Celes varabilis* female, b) *C. varabilis* male, c*) Dectius verrucivorus* female, d) *D. verrucivorus* male, e) *Melanogryllus desertus* female, f) *M. desertus* male, g) *Paracyptera labiata* female, h) *P. labiata* male.

Thermogravimetric analysis results of the chitins isolated from grasshoppers are given in Fig. 2. It was found that the thermograms of all species have two decomposition steps. The mass loss in the first step occurred in the range of 0–150°C due to the evaporation of water. The second step occurred in the range of 150–400°C, because of degradation of the chitin molecules. The weight loss in the first step was 3–6%, while in the second step it was 73–96%, according to species. Two steps of mass losses were also observed by other researchers [2, 6, 38]. The most decomposed (DTG_max_) temperature was found to be around 387°C (385–390°C). In our work, results of DTG_max_ were similar to other studies, where DTG_max_ was reported to be changing from 350 to 387°C [1, 2, 5, 6, 10, 38, 39]. Results of thermogram analysis showed no significant differences between female and male.

DTG_max_ values of the male and females were highly similar. But some differences in total mass loss and in amount of the non-decomposed material were observed. Particularly, total mass losses of chitin samples from females were found to be higher than males (Fig. 2). Similarly, ash contents of chitin from females were lower than the males. Very high decomposition rate (99%) was observed in male and female from *M. desertus*. This could be useful in further chitin application studies.

### XRD

**Figure 3:**
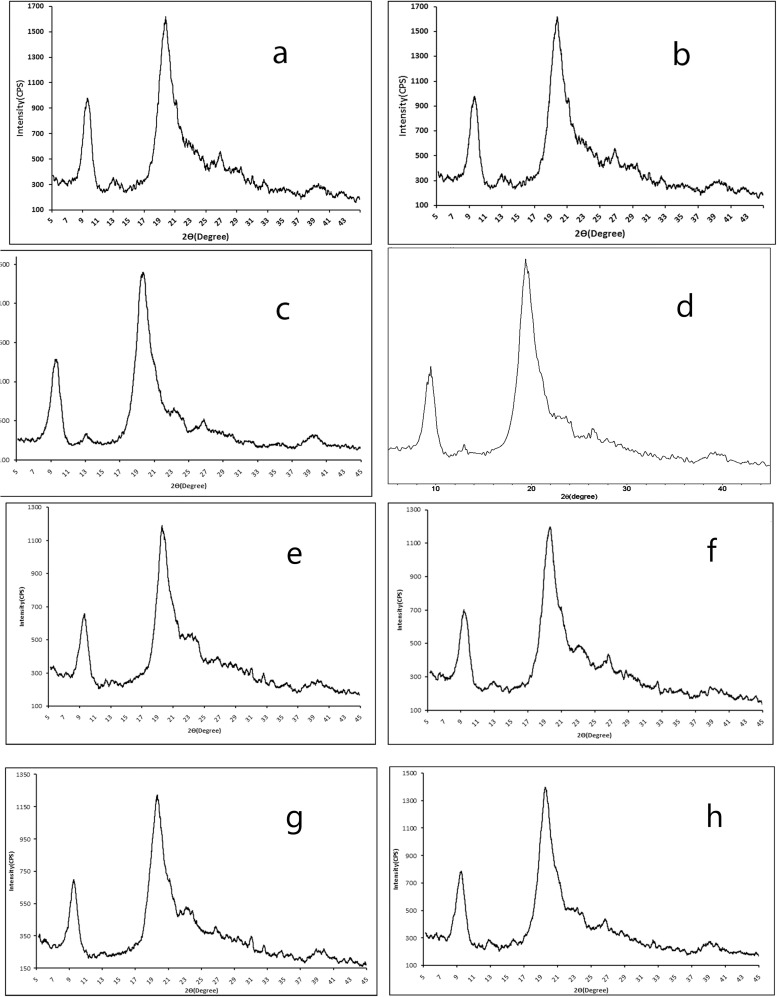
XRD of chitin extracted from female and male grasshoppers. a) *Celes varabilis* female, b) *C. varabilis* male, c) *Dectius verrucivorus* male, d) *Melanogryllus desertus* female, e) *M. desertus* male, f) *Paracyptera labiata* female, g) *P. labiata* male.

To detect the crystallinity of the isolated chitin from grasshoppers, female and male X-ray diffractometry analyse was applied. From Fig. 3, all chitin samples isolated from grasshoppers, showed similar XRD patterns. In the spectrum of X-ray diffraction two sharp peaks (around 9° and 19°) and four weak peaks (around 12°, 21°, 23°, 26°) were observed. In previous studies, in X-ray diffractometry spectrum, similar peaks were observed in alpha chitin isolated from insects, crabs, shrimp, fungi and anthozoan [2, 6, 7, 38]. This shows that chitin isolated from grasshoppers is also in alpha form.

The crystalline index (CrI), calculated from the X-ray diffraction data, are presented in S1 Table. In our study, chitin structures of grasshoppers’ CrI values are changing from 75 to 80%. Other studies showed that CrI values are changing from 43 to 91.7% in different organisms. For example, in fungi CrI values ranging from 43.6 to 80% [4], larvae cuticles and silkworm pupa exuviae (*Bombyx mori*) CrI values were between 54 and 58% [40], CrI values of chitins extracted from cicada slough, rice-field crab shell and shrimp were recorded at around 90% [2, 6]. Crystallinity of grasshopper chitin, compared with other organisms, is average.

Al-Sagheer et al. [26] observed that the male crab is more crystalline than the female crab. Our data shows that for females and males of the same species, CrI values are practically the same. Although, male chitin of *P. labiata* is lightly more crystalline than female chitin (79 and 74% respectively), the opposite result was observed in *C. variabilis*, where male CrI values were lower than female (76 and 80% respectively).

### SEM

**Figure 4:**
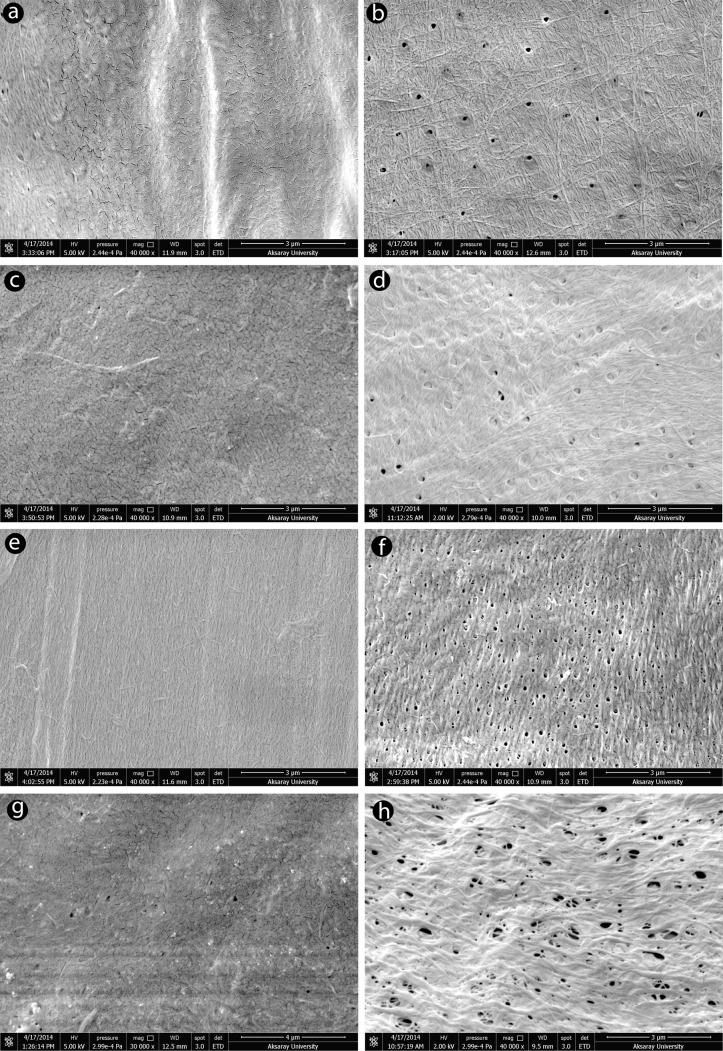
SEM of four grasshoppers spiecies: a) *Celes variabilis* female, b) *C. variabilis* male, c) *Decticus verrucivorus* female, d) *D. verrucivorus* male, e) *Melanogryllus desertus* female, f) *M. desertus* male, g) *Paracyptera labiate* female, h) *P. labiate* male.

To better understand chitin surface morphology, the samples were examined using a scanning electron microscope. Fig. 4 presents SEM images of grasshoppers’ female and male chitin surface structures. The chitin surfaces obtained from the grasshopper males were found to be porous with highly adherent nanofibers. The diameters of the pores were 180–260nm, and the widths of the nanofibers were between 25 and 80nm (detailed information about pores and nanofiber sizes are shown in Table 5). The female grasshoppers’ chitin was expected to be similar to that of the males, but the surface structure was dissimilar. The surface of the chitin isolated from the females resembled a dried land surface. The females’ chitin surface morphology did not show any porosity structure, while nanofibers were observed only in *M. desertus*. The same chemical procedures were performed to obtain chitin from four grasshopper species, both female and male, but the surface morphology was observed to be greatly different between the females and males.

**Table 5:** Pores and fibers sizes of chitin samples isolated from four male and female orthopteran species.

**Species**	**Pore size (nm)**		**Fiber size (nm)**	
	**Female**	**Male**	**Female**	**Male**
*Celes variabilis*	No pores	180–200	No fibers	30–60
*Decticus verrucivorus*	No pores	200–250	No fibers	25–35
*Melanogryllus desertus*	No pores	90–100	20–35	80–90
*Paracyptera labiata*	No pores	180–250	No fibers	70–85

## Conclusions

This is the first study to report differences in the chitin extracted from different sexes of grasshoppers of the same species. Characterization of chitin was carried out with FT-IR, EA, XRD, TGA, and SEM. The results from FT-IR, EA, XRD, and TGA showed that the chitin under investigation was in alpha form. These analyses did not show significant differences between female and male chitins, but chitin content was determined to be higher in males than females. Additionally, enzymatic digestion of the grasshoppers’ chitins and commercial chitin was performed using chitinase from *Streptomyces griseus.* Potassium ferricyanide assay of the products released during the enzymatic digestion suggested that there were small differences in the quantity of the hydrolytic products of the extracted chitins from either sex or the commercial chitin. The grasshoppers’ chitins showed high reducing sugar production rates close to the commercial chitin under the studied conditions. The results of the SEM analysis showed considerable dissimilarity between the sexes. The chitin surface of female grasshoppers resembled dry land (cracked and scaly); no nanopores were observed, whereas nanofibers were present only in *M. desertus*. Both nanopores and nanofibers were observed in the chitin of male grasshoppers. These dissimilarities suggest that chitin samples obtained from the female and male could be used in different fields of medicine, biotechnology, wastewater treatment, or agriculture, depending on gender. The alpha chitin obtained from grasshoppers has the potential to be used as an alternative or new source of chitin and its products.

## Supporting Information

S1 Table Results of X-ray diffraction and crystalline index values. (DOC)
