# Supplementary material for: Differentiations of Chitin Content and Surface Morphologies of Chitins Extracted from Male and Female Grasshopper Species
Source: PLoS One. 2015 Jan 30;10(1):e0115531. doi: 10.1371/journal.pone.0115531 (PMC4312026; doi:10.1371/journal.pone.0115531)
Supplement: S1 Table — (DOC) [file pone.0115531.s001.doc]

**Table .** Results of X-ray diffraction and crystalline index values

| **Species** | **XRD Peaks** | **CrI (%)** |
| --- | --- | --- |
| *Celes variabilis*(female) | 9.26, 12.72, 19.56, 21.16, 23.38, 26.44 | 80 |
| *Celes variabilis*(male) | 9.5, 12.91, 19.68, 21.12, 22.76, 26.66 | 76 |
| *Decticus verrucivorus*(female) | 9.42, 13.22, 19.38, 21.84, 23.64, 26.5 | 80 |
| *Decticus verrucivorus*(male) | 9.48, 12.9, 19.4, 21.02, 23.16, 26.74 | 80 |
| *Melanogryllus desertus*(female) | 9.38, 12.42, 19.56, 21.5, 23.14, 26.72 | 78 |
| *Melanogryllus desertus*(male) | 9.42, 12.74, 19.54, 21.03, 23.72, 26.56 | 77 |
| *Paracyptera labiata*(female) | 9.28, 12.76, 19.62, 21.12, 23.48, 26.06 | 75 |
| *Paracyptera labiata*(male) | 9.34, 12.72, 19.66, 21.26, 23.12, 26.36 | 79 |
